# 1,10-Phenanthrolin-1-ium nitrate–aqua­bis­(4-hydroxy­benzoato-κ^2^ *O*,*O*′)(nitrato-κ^2^ *O*,*O*′)(1,10-phenanthroline-κ^2^ *N*,*N*′)erbium(III)–1,10-phenanthroline–water (1/1/0.5/2)

**DOI:** 10.1107/S1600536810051767

**Published:** 2010-12-15

**Authors:** Fwu Ming Shen, Shie Fu Lush

**Affiliations:** a Department of Biotechnology, Yuanpei University, HsinChu 30015, Taiwan; b Department of Genernal Eduction Center, Yuanpei University, HsinChu 30015, Taiwan

## Abstract

In the title compound, C_12_H_9_N_2_^+^·NO_3_^−^·[Er(C_7_H_5_O_3_)_2_(NO_3_)(C_12_H_8_N_2_)(H_2_O)]·0.5C_12_H_8_N_2_·2H_2_O, the water-mol­ecule-coordinated Er^III^ ion is chelated by one 1,10-phenanthroline (phen) ligand, two 4-hy­droxy­benzoate anions and one nitrate anion in a monocapped square-anti­prismatic coordination geometry. The uncoordinating phen mol­ecule is approximately parallel to the 1,10-phenanthrolin-1-ium (Hphen) anion [dihedral angle = 3.3 (4)°]. The centroid–centroid distance of 3.801 (5) Å between pyridine rings suggests the existence of π–π stacking. The crystal structure contains an extensive network of classical O—H⋯O and N—H⋯O and weak C—H⋯O hydrogen bonds. C—H⋯π inter­actions between phen and 4-hy­droxy­benzoate is also present in the crystal structure. In the crystal, the uncoordinating phen is equally disordered over two sites about an inversion center.

## Related literature

For a related hydro­thermal substitution reaction, see: Xiong *et al.* (2001 ▶). For related structures, see: Liu *et al.* (2007 ▶, 2010 ▶); Neelgund *et al.* (2007 ▶).
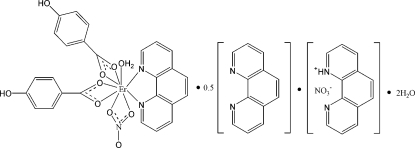


## Experimental

### 

#### Crystal data

C_12_H_9_N_2_^+^·NO_3_^−^·[Er(C_7_H_5_O_3_)_2_(NO_3_)(C_12_H_8_N_2_)(H_2_O)]·0.5C_12_H_8_N_2_·2H_2_O
*M* *_r_* = 1071.07
Triclinic, 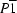

*a* = 10.9464 (2) Å
*b* = 11.3682 (3) Å
*c* = 19.2638 (5) Å
α = 77.108 (2)°
β = 84.790 (2)°
γ = 67.250 (2)°
*V* = 2154.95 (10) Å^3^
*Z* = 2
Mo *K*α radiation
μ = 2.03 mm^−1^
*T* = 97 K
0.35 × 0.20 × 0.18 mm

#### Data collection

Oxford Diffraction Gemini-S CCD diffractometer
Absorption correction: multi-scan (*CrysAlis PRO*; Oxford Diffraction, 2009 ▶) *T*_min_ = 0.629, *T*_max_ = 0.694
16237 measured reflections
7710 independent reflections
6632 reflections with *I* > 2σ(*I*)
*R*_int_ = 0.028

#### Refinement

*R*[*F* ^2^ > 2σ(*F* ^2^)] = 0.038
*wR*(*F* ^2^) = 0.105
*S* = 1.05
7710 reflections
583 parameters
H-atom parameters constrained
Δρ_max_ = 2.48 e Å^−3^
Δρ_min_ = −1.41 e Å^−3^

### 

Data collection: *CrysAlis CCD* (Oxford Diffraction, 2008 ▶); cell refinement: *CrysAlis RED* (Oxford Diffraction, 2008 ▶); data reduction: *CrysAlis RED*; program(s) used to solve structure: *SHELXS97* (Sheldrick, 2008 ▶); program(s) used to refine structure: *SHELXL97* (Sheldrick, 2008 ▶); molecular graphics: *ORTEP-3* (Farrugia, 1997 ▶); software used to prepare material for publication: *PLATON* (Spek, 2009 ▶).

## Supplementary Material

Crystal structure: contains datablocks global, I. DOI: 10.1107/S1600536810051767/xu5103sup1.cif

Structure factors: contains datablocks I. DOI: 10.1107/S1600536810051767/xu5103Isup2.hkl

Additional supplementary materials:  crystallographic information; 3D view; checkCIF report

## Figures and Tables

**Table 1:** Selected bond lengths (Å)

Er1—O1	2.470 (4)
Er1—O2	2.376 (4)
Er1—O3*W*	2.358 (3)
Er1—O4	2.372 (3)
Er1—O5	2.399 (3)
Er1—O6	2.366 (4)
Er1—O7	2.433 (4)
Er1—N1	2.461 (4)
Er1—N2	2.489 (4)

**Table 2:** Hydrogen-bond geometry (Å, °) *Cg*4 is the centroid of the C32–C37 ring.

*D*—H⋯*A*	*D*—H	H⋯*A*	*D*⋯*A*	*D*—H⋯*A*
O1*W*—H1*A*⋯O2^i^	0.82	2.55	3.270 (6)	148
O1*W*—H1*A*⋯O6^i^	0.82	2.22	2.875 (6)	137
O1*W*—H1*B*⋯O12^i^	0.82	2.06	2.872 (10)	172
O2*W*—H2*A*⋯O9^ii^	0.82	1.94	2.737 (8)	164
O2*W*—H2*B*⋯O11^i^	0.82	2.01	2.789 (10)	157
O3*W*—H3*A*⋯O5^iii^	0.82	1.90	2.671 (5)	155
O3*W*—H3*B*⋯O7^iii^	0.82	2.16	2.836 (5)	140
N4—H4⋯O2*W*	0.86	1.91	2.725 (9)	157
O8—H8⋯O1*W*^iv^	0.82	1.87	2.659 (6)	160
O9—H9⋯O11^v^	0.82	2.09	2.803 (12)	145
O9—H9⋯O12^v^	0.82	2.43	3.172 (12)	151
C34—H34⋯O3^v^	0.93	2.43	3.256 (8)	148
C48—H48⋯O12^vi^	0.93	2.27	3.046 (11)	141
C62—H62⋯O1^vii^	0.93	2.55	3.387 (10)	150
C70—H70⋯O13^i^	0.93	2.35	3.244 (13)	161
C83—H83⋯*Cg*4^ii^	0.93	2.78	3.628 (9)	152

Symmetry codes: (i) 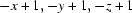; (ii) 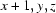; (iii) 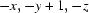; (iv) 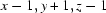; (v) 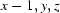; (vi) 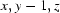; (vii) 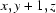.

**Fig. 1.**
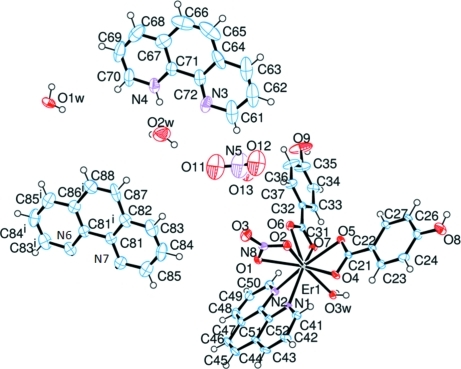
View of the title compound with the atom numbering scheme. Displacement ellipsoids for non-H atoms are drawn at the 50% probability level [symmetry code: (i) 2 - x, -y, 1 - z].

**Fig. 2.**
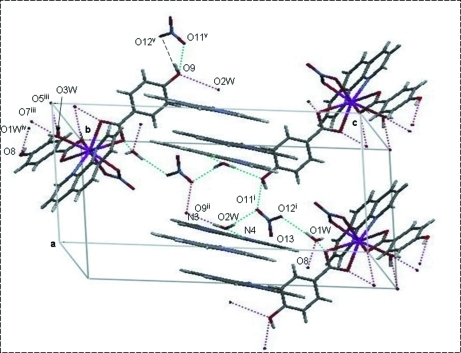
The molecular packing for the title compound. Hydrogen bonds are shown as dashed lines.

## supplementary crystallographic information

### Comment

The coordination chemistry of erbium (III) with N and O donor ligands has been investigated in the past decade and numbers of erbium (III) complexes with different donor ligands have been synthesized and studied by X-ray crystallography (Liu *et al.*, 2010; Neelgund *et al.*, 2007; Liu *et al.*, 2007). The title compound was recently obtained from the reaction of erbium nitrate, sodium benzoate and phen in an methanol-water mixture, and its crystal structure is reported here. Since no 4-hydrobenzoic acid ligand is present in the starting reaction mixture, it may be derived from the benzoic acid *via in situ* substitution(Xiong *et al.*, 2001) under hydrothermal condition.

The Er^III^ ion is nine-coordinated by two N atoms of a phen ligand, four carboxylate O atoms of two 4-hydroxybenzoate anions, two O atoms of nitrate anion and one O atom of a water molecule. The resulting coordination geometry is a monocapped square antiprismatic coordination (Table 1 and Fig. 1).

The phen molecule is approximately parallel to 1,10-phenanthrolinium (Hphen), making dihedral angle of 3.3 (4)°.The centroid-centroid distance between N4-pyridine and N7-pyridine rings is 3.801 (5) Å, indictative of π—π interaction.The crystal structure contain an extensive network of classical (O—H···O, N—H···O) and weak (C—H···O) hydrogen bonds (Table 2 and Fig. 2).

In addition, C—H···π interaction (C83—H83···*Cg*4(C32—C37); full details and symmetry code are given in Table 2.) between phen and 4-hydroxybenzoate is present in the crystal structure.

### Experimental

Erbium trinitrate solution was prepared by dissolving Er(NO_3_)_3_.6H_2_O (0.4631 g, 1.00 mmole) at room temperature with stirring. The ligand solution was prepared by dissolving benzoic acid (0.4889 g, 4 mmole) and 1,10-phenanthroline (4 mmole) in 20 ml methanol at room temperature. The pH of the ligand solution was adjusted to about 6 with 2 N NaOH. The Er solution was added drop wise and slowly to the ligand solution. The reaction mixture was stirred for 2 h at room temperature. Pink crystals were obtained at room temperature over a period 3 months.

### Refinement

Position C82, N6, C86, N7, C87 and C88 of the phen ring split into two different atoms with 50% occupancies for each, respectively. H atoms bonded to O and N atoms were placed in calculated positions and refined with the distances constrains of O—H = 0.82, N—H = 0.86 Å, and *U*_iso_(H)= 1.2*U*_eq_(N) and 1.5*U*_eq_(O). Other H atoms were positioned geometrically with C—H = 0.93 Å and refined using a riding model with *U*_iso_(H) = 1.2*U*_eq_(C).

### Crystal data

**Table:**

C_12_H_9_N_2_^+^·NO_3_^−^·[Er(C_7_H_5_O_3_)_2_(NO_3_)(C_12_H_8_N_2_)(H_2_O)]·0.5C_12_H_8_N_2_·2H_2_O	*Z* = 2
*M**_r_* = 1071.07	*F*(000) = 1076
Triclinic, *P*1	*D*_x_ = 1.651 Mg m^−^^3^
Hall symbol: -P 1	Mo *K*α radiation, λ = 0.71073 Å
*a* = 10.9464 (2) Å	Cell parameters from 12670 reflections
*b* = 11.3682 (3) Å	θ = 2.4–29.2°
*c* = 19.2638 (5) Å	µ = 2.03 mm^−^^1^
α = 77.108 (2)°	*T* = 97 K
β = 84.790 (2)°	Block, pink
γ = 67.250 (2)°	0.35 × 0.20 × 0.18 mm
*V* = 2154.95 (10) Å^3^	

### Data collection

**Table:**

Oxford Diffraction Gemini-S CCD diffractometer	7710 independent reflections
Radiation source: fine-focus sealed tube	6632 reflections with *I* > 2σ(*I*)
graphite	*R*_int_ = 0.028
Detector resolution: 9 pixels mm^-1^	θ_max_ = 25.2°, θ_min_ = 2.5°
ω scans	*h* = −13→13
Absorption correction: multi-scan (*CrysAlis PRO*; Oxford Diffraction, 2009)	*k* = −13→11
*T*_min_ = 0.629, *T*_max_ = 0.694	*l* = −23→22
16237 measured reflections	

### Refinement

**Table:**

Refinement on *F*^2^	Primary atom site location: structure-invariant direct methods
Least-squares matrix: full	Secondary atom site location: difference Fourier map
*R*[*F*^2^ > 2σ(*F*^2^)] = 0.038	Hydrogen site location: inferred from neighbouring sites
*wR*(*F*^2^) = 0.105	H-atom parameters constrained
*S* = 1.05	*w* = 1/[σ^2^(*F*_o_^2^) + (0.0656*P*)^2^ + 2.9468*P*] where *P* = (*F*_o_^2^ + 2*F*_c_^2^)/3
7710 reflections	(Δ/σ)_max_ = 0.001
583 parameters	Δρ_max_ = 2.48 e Å^−^^3^
0 restraints	Δρ_min_ = −1.41 e Å^−^^3^

### Special details

**Table:**

Geometry. Bond distances, angles *etc*. have been calculated using the rounded fractional coordinates. All su's are estimated from the variances of the (full) variance-covariance matrix. The cell e.s.d.'s are taken into account in the estimation of distances, angles and torsion angles
Refinement. Refinement on *F*^2^ for ALL reflections except those flagged by the user for potential systematic errors. Weighted *R*-factors *wR* and all goodnesses of fit *S* are based on *F*^2^, conventional *R*-factors *R* are based on *F*, with *F* set to zero for negative *F*^2^. The observed criterion of *F*^2^ > σ(*F*^2^) is used only for calculating -*R*-factor-obs *etc*. and is not relevant to the choice of reflections for refinement. *R*-factors based on *F*^2^ are statistically about twice as large as those based on *F*, and *R*-factors based on ALL data will be even larger.

### Fractional atomic coordinates and isotropic or equivalent isotropic displacement parameters (Å^2^)

**Table:**

	*x*	*y*	*z*	*U*_iso_*/*U*_eq_	Occ. (<1)
Er1	0.23247 (2)	0.40305 (2)	0.07892 (1)	0.0145 (1)	
O1	0.3934 (4)	0.3029 (4)	0.17753 (19)	0.0232 (11)	
O2	0.3711 (3)	0.4927 (4)	0.11782 (19)	0.0234 (11)	
O3	0.4913 (4)	0.4118 (5)	0.2139 (2)	0.0406 (16)	
O3W	0.1453 (3)	0.3643 (3)	−0.01739 (18)	0.0176 (10)	
O4	0.2763 (3)	0.5363 (3)	−0.02595 (18)	0.0199 (11)	
O5	0.0975 (3)	0.6273 (3)	0.03472 (18)	0.0176 (11)	
O6	0.1303 (3)	0.4721 (4)	0.18419 (18)	0.0204 (11)	
O7	0.0106 (3)	0.4202 (3)	0.12003 (18)	0.0178 (11)	
O8	0.0969 (4)	1.1108 (4)	−0.2073 (2)	0.0379 (14)	
O9	−0.4015 (6)	0.6324 (7)	0.3624 (3)	0.084 (3)	
N1	0.4400 (4)	0.2592 (4)	0.0322 (2)	0.0166 (12)	
N2	0.2730 (4)	0.1678 (4)	0.1217 (2)	0.0200 (12)	
N8	0.4204 (4)	0.4033 (5)	0.1714 (2)	0.0224 (16)	
C21	0.1771 (5)	0.6361 (5)	−0.0180 (3)	0.0165 (16)	
C22	0.1524 (5)	0.7613 (5)	−0.0678 (3)	0.0165 (16)	
C23	0.2377 (5)	0.7697 (5)	−0.1251 (3)	0.0209 (17)	
C24	0.2173 (6)	0.8873 (6)	−0.1703 (3)	0.0265 (17)	
C25	0.1121 (5)	0.9991 (5)	−0.1603 (3)	0.0240 (17)	
C26	0.0270 (5)	0.9913 (5)	−0.1026 (3)	0.0204 (17)	
C27	0.0462 (5)	0.8744 (5)	−0.0570 (3)	0.0189 (16)	
C31	0.0201 (5)	0.4661 (5)	0.1733 (3)	0.0173 (17)	
C32	−0.0930 (5)	0.5123 (5)	0.2214 (3)	0.0188 (17)	
C33	−0.2127 (5)	0.5001 (5)	0.2132 (3)	0.0233 (17)	
C34	−0.3172 (6)	0.5408 (6)	0.2595 (3)	0.0302 (19)	
C35	−0.3038 (7)	0.5950 (8)	0.3140 (4)	0.048 (3)	
C36	−0.1851 (7)	0.6091 (8)	0.3221 (4)	0.048 (3)	
C37	−0.0815 (6)	0.5674 (6)	0.2763 (3)	0.0307 (19)	
C41	0.5172 (5)	0.3009 (5)	−0.0162 (3)	0.0196 (16)	
C42	0.6408 (5)	0.2174 (6)	−0.0376 (3)	0.0232 (16)	
C43	0.6854 (5)	0.0893 (5)	−0.0062 (3)	0.0234 (16)	
C44	0.6101 (5)	0.0409 (5)	0.0460 (3)	0.0226 (17)	
C45	0.6523 (6)	−0.0923 (6)	0.0828 (3)	0.0282 (17)	
C46	0.5745 (6)	−0.1344 (6)	0.1304 (3)	0.0323 (19)	
C47	0.4424 (6)	−0.0499 (5)	0.1450 (3)	0.0247 (17)	
C48	0.3541 (6)	−0.0925 (6)	0.1906 (3)	0.0315 (19)	
C49	0.2285 (6)	−0.0087 (6)	0.1985 (3)	0.0282 (17)	
C50	0.1909 (5)	0.1214 (6)	0.1633 (3)	0.0241 (17)	
C51	0.3980 (5)	0.0820 (5)	0.1119 (3)	0.0186 (17)	
C52	0.4844 (5)	0.1306 (5)	0.0621 (3)	0.0176 (16)	
N6	0.9403 (6)	0.1675 (6)	0.4488 (3)	0.0379 (19)	0.500
N7	0.9737 (6)	−0.0343 (6)	0.4147 (3)	0.0373 (19)	0.500
C81	0.9773 (6)	0.0350 (6)	0.4644 (3)	0.0307 (17)	
C82	0.9403 (6)	0.1675 (6)	0.4488 (3)	0.0379 (19)	0.500
C83	0.8975 (7)	0.2307 (8)	0.3798 (4)	0.050 (3)	
C84	0.8912 (8)	0.1665 (9)	0.3303 (4)	0.054 (3)	
C85	0.9291 (7)	0.0356 (9)	0.3474 (4)	0.050 (3)	
C86	0.9737 (6)	−0.0343 (6)	0.4147 (3)	0.0373 (19)	0.500
C87	0.9379 (12)	0.2441 (15)	0.5036 (8)	0.049 (5)	0.500
C88	0.9813 (13)	0.1792 (15)	0.5675 (8)	0.045 (5)	0.500
N3	0.6566 (5)	0.8871 (7)	0.4088 (3)	0.047 (2)	
N4	0.7372 (5)	0.7810 (6)	0.5468 (3)	0.0404 (19)	
C61	0.6104 (7)	0.9421 (10)	0.3441 (4)	0.075 (2)	
C62	0.5602 (7)	1.0749 (10)	0.3179 (5)	0.075 (2)	
C63	0.5605 (7)	1.1543 (11)	0.3588 (5)	0.075 (2)	
C64	0.6096 (6)	1.1019 (8)	0.4282 (4)	0.053 (3)	
C65	0.6144 (8)	1.1804 (9)	0.4766 (6)	0.075 (4)	
C66	0.6588 (9)	1.1252 (10)	0.5436 (6)	0.072 (4)	
C67	0.7030 (7)	0.9884 (9)	0.5694 (4)	0.049 (3)	
C68	0.7458 (8)	0.9284 (12)	0.6377 (4)	0.069 (4)	
C69	0.7839 (8)	0.7975 (12)	0.6597 (4)	0.072 (4)	
C70	0.7774 (7)	0.7261 (9)	0.6133 (4)	0.056 (3)	
C71	0.7003 (5)	0.9088 (7)	0.5234 (3)	0.0334 (19)	
C72	0.6538 (6)	0.9681 (7)	0.4511 (3)	0.041 (2)	
O11	0.4009 (8)	0.5285 (9)	0.3933 (4)	0.1090 (19)	
O12	0.3154 (8)	0.6897 (9)	0.3035 (4)	0.1090 (19)	
O13	0.2058 (8)	0.5725 (9)	0.3489 (4)	0.1090 (19)	
N5	0.3076 (11)	0.6002 (11)	0.3496 (6)	0.1090 (19)	
O1W	0.8550 (4)	0.2838 (4)	0.8087 (2)	0.0373 (14)	
O2W	0.7115 (6)	0.5915 (6)	0.4911 (3)	0.070 (2)	
H3A	0.07580	0.35180	−0.01090	0.0260*	
H3B	0.13890	0.42290	−0.05230	0.0260*	
H8	0.03190	1.17010	−0.19610	0.0560*	
H9	−0.46530	0.61790	0.35310	0.1260*	
H23	0.30870	0.69550	−0.13270	0.0250*	
H24	0.27510	0.89200	−0.20830	0.0320*	
H26	−0.04340	1.06600	−0.09500	0.0240*	
H27	−0.01140	0.87000	−0.01880	0.0220*	
H33	−0.22230	0.46410	0.17610	0.0280*	
H34	−0.39630	0.53160	0.25390	0.0360*	
H36	−0.17620	0.64680	0.35860	0.0580*	
H37	−0.00240	0.57640	0.28220	0.0370*	
H41	0.48810	0.38930	−0.03680	0.0240*	
H42	0.69090	0.24950	−0.07260	0.0280*	
H43	0.76710	0.03300	−0.01960	0.0280*	
H45	0.73580	−0.15070	0.07320	0.0340*	
H46	0.60650	−0.22060	0.15490	0.0390*	
H48	0.38160	−0.17850	0.21540	0.0380*	
H49	0.16820	−0.03750	0.22700	0.0340*	
H50	0.10450	0.17830	0.16920	0.0290*	
H83	0.87210	0.32070	0.36750	0.0600*	
H84	0.86100	0.21180	0.28470	0.0650*	
H85	0.92510	−0.00870	0.31300	0.0590*	
H87	0.90650	0.33460	0.49240	0.0580*	0.500
H88	0.98420	0.22370	0.60200	0.0540*	0.500
H4	0.73500	0.73310	0.51850	0.0480*	
H61	0.61150	0.88810	0.31400	0.0890*	
H62	0.52650	1.10840	0.27200	0.0890*	
H63	0.52850	1.24390	0.34160	0.0890*	
H65	0.58650	1.27020	0.46130	0.0890*	
H66	0.66070	1.17790	0.57380	0.0860*	
H68	0.74870	0.97820	0.66940	0.0820*	
H69	0.81410	0.75720	0.70580	0.0860*	
H70	0.80180	0.63660	0.62860	0.0670*	
H1A	0.82320	0.35200	0.82330	0.0560*	
H1B	0.80360	0.28640	0.77950	0.0560*	
H2A	0.68500	0.58770	0.45350	0.1050*	
H2B	0.69560	0.53770	0.52280	0.1050*	

### Atomic displacement parameters (Å^2^)

**Table:**

	*U*^11^	*U*^22^	*U*^33^	*U*^12^	*U*^13^	*U*^23^
Er1	0.0110 (1)	0.0126 (1)	0.0177 (1)	−0.0017 (1)	−0.0013 (1)	−0.0031 (1)
O1	0.0243 (19)	0.018 (2)	0.026 (2)	−0.0047 (16)	−0.0082 (16)	−0.0046 (16)
O2	0.0178 (18)	0.026 (2)	0.023 (2)	−0.0060 (16)	−0.0008 (15)	−0.0020 (16)
O3	0.039 (2)	0.045 (3)	0.045 (3)	−0.017 (2)	−0.019 (2)	−0.014 (2)
O3W	0.0143 (17)	0.0174 (19)	0.0191 (18)	−0.0043 (15)	−0.0005 (14)	−0.0025 (15)
O4	0.0135 (17)	0.0150 (19)	0.025 (2)	0.0005 (15)	0.0031 (14)	−0.0037 (15)
O5	0.0125 (17)	0.0163 (19)	0.0219 (19)	−0.0042 (14)	0.0000 (14)	−0.0022 (15)
O6	0.0158 (18)	0.024 (2)	0.0207 (19)	−0.0077 (16)	−0.0009 (14)	−0.0024 (15)
O7	0.0178 (18)	0.0184 (19)	0.0170 (18)	−0.0071 (15)	0.0021 (14)	−0.0039 (15)
O8	0.047 (3)	0.019 (2)	0.035 (2)	−0.0078 (19)	0.015 (2)	0.0048 (18)
O9	0.058 (4)	0.148 (7)	0.097 (5)	−0.067 (4)	0.056 (3)	−0.096 (5)
N1	0.012 (2)	0.016 (2)	0.022 (2)	−0.0026 (17)	−0.0039 (17)	−0.0077 (18)
N2	0.019 (2)	0.019 (2)	0.023 (2)	−0.0070 (19)	−0.0034 (18)	−0.0052 (19)
N8	0.012 (2)	0.028 (3)	0.028 (3)	−0.004 (2)	−0.0001 (19)	−0.014 (2)
C21	0.014 (2)	0.017 (3)	0.018 (3)	−0.004 (2)	−0.003 (2)	−0.005 (2)
C22	0.013 (2)	0.014 (3)	0.023 (3)	−0.005 (2)	−0.003 (2)	−0.004 (2)
C23	0.019 (3)	0.020 (3)	0.022 (3)	−0.006 (2)	0.001 (2)	−0.004 (2)
C24	0.030 (3)	0.028 (3)	0.022 (3)	−0.013 (3)	0.011 (2)	−0.007 (2)
C25	0.028 (3)	0.019 (3)	0.024 (3)	−0.010 (2)	−0.001 (2)	0.000 (2)
C26	0.019 (3)	0.012 (3)	0.027 (3)	−0.001 (2)	−0.001 (2)	−0.006 (2)
C27	0.014 (2)	0.020 (3)	0.021 (3)	−0.005 (2)	0.004 (2)	−0.005 (2)
C31	0.021 (3)	0.012 (3)	0.017 (3)	−0.006 (2)	−0.002 (2)	0.001 (2)
C32	0.019 (3)	0.015 (3)	0.020 (3)	−0.006 (2)	0.001 (2)	0.000 (2)
C33	0.027 (3)	0.014 (3)	0.027 (3)	−0.007 (2)	0.001 (2)	−0.002 (2)
C34	0.023 (3)	0.034 (3)	0.040 (4)	−0.017 (3)	0.010 (3)	−0.013 (3)
C35	0.041 (4)	0.068 (5)	0.055 (4)	−0.035 (4)	0.027 (3)	−0.038 (4)
C36	0.049 (4)	0.078 (6)	0.044 (4)	−0.042 (4)	0.023 (3)	−0.041 (4)
C37	0.030 (3)	0.041 (4)	0.029 (3)	−0.020 (3)	0.008 (2)	−0.014 (3)
C41	0.016 (2)	0.020 (3)	0.025 (3)	−0.007 (2)	−0.002 (2)	−0.008 (2)
C42	0.013 (2)	0.027 (3)	0.033 (3)	−0.008 (2)	0.003 (2)	−0.013 (2)
C43	0.013 (2)	0.023 (3)	0.036 (3)	−0.001 (2)	−0.001 (2)	−0.020 (3)
C44	0.015 (3)	0.018 (3)	0.035 (3)	0.000 (2)	−0.009 (2)	−0.014 (2)
C45	0.023 (3)	0.018 (3)	0.039 (3)	0.002 (2)	−0.009 (3)	−0.011 (3)
C46	0.037 (3)	0.014 (3)	0.039 (4)	0.001 (3)	−0.013 (3)	−0.006 (3)
C47	0.033 (3)	0.015 (3)	0.025 (3)	−0.007 (2)	−0.010 (2)	−0.002 (2)
C48	0.049 (4)	0.018 (3)	0.028 (3)	−0.014 (3)	−0.013 (3)	0.002 (2)
C49	0.038 (3)	0.025 (3)	0.026 (3)	−0.017 (3)	−0.002 (2)	−0.004 (2)
C50	0.025 (3)	0.028 (3)	0.022 (3)	−0.012 (2)	−0.002 (2)	−0.006 (2)
C51	0.020 (3)	0.017 (3)	0.019 (3)	−0.005 (2)	−0.007 (2)	−0.005 (2)
C52	0.015 (2)	0.017 (3)	0.023 (3)	−0.005 (2)	−0.006 (2)	−0.008 (2)
N6	0.031 (3)	0.033 (3)	0.045 (4)	−0.013 (3)	0.004 (3)	0.001 (3)
N7	0.031 (3)	0.047 (4)	0.036 (3)	−0.019 (3)	0.005 (2)	−0.007 (3)
C81	0.026 (3)	0.034 (3)	0.030 (3)	−0.013 (3)	0.006 (2)	−0.002 (3)
C82	0.031 (3)	0.033 (3)	0.045 (4)	−0.013 (3)	0.004 (3)	0.001 (3)
C83	0.048 (4)	0.040 (4)	0.056 (5)	−0.022 (4)	0.001 (4)	0.014 (4)
C84	0.055 (5)	0.068 (6)	0.037 (4)	−0.033 (4)	−0.011 (3)	0.016 (4)
C85	0.038 (4)	0.080 (6)	0.037 (4)	−0.032 (4)	0.004 (3)	−0.008 (4)
C86	0.031 (3)	0.047 (4)	0.036 (3)	−0.019 (3)	0.005 (2)	−0.007 (3)
C87	0.022 (6)	0.047 (9)	0.054 (9)	−0.005 (6)	−0.003 (6)	0.022 (7)
C88	0.032 (7)	0.049 (9)	0.053 (9)	−0.016 (7)	0.006 (6)	−0.011 (7)
N3	0.036 (3)	0.070 (5)	0.027 (3)	−0.016 (3)	0.000 (2)	0.001 (3)
N4	0.029 (3)	0.050 (4)	0.030 (3)	−0.008 (3)	0.000 (2)	0.003 (3)
C61	0.029 (2)	0.106 (5)	0.052 (3)	−0.012 (3)	0.003 (2)	0.030 (3)
C62	0.029 (2)	0.106 (5)	0.052 (3)	−0.012 (3)	0.003 (2)	0.030 (3)
C63	0.029 (2)	0.106 (5)	0.052 (3)	−0.012 (3)	0.003 (2)	0.030 (3)
C64	0.022 (3)	0.043 (5)	0.066 (5)	−0.001 (3)	0.013 (3)	0.016 (4)
C65	0.046 (5)	0.043 (5)	0.120 (9)	−0.013 (4)	0.040 (5)	−0.010 (6)
C66	0.056 (5)	0.061 (6)	0.112 (8)	−0.032 (5)	0.043 (6)	−0.044 (6)
C67	0.028 (3)	0.071 (6)	0.054 (5)	−0.020 (4)	0.013 (3)	−0.026 (4)
C68	0.040 (4)	0.126 (10)	0.045 (5)	−0.027 (5)	0.005 (4)	−0.038 (6)
C69	0.038 (4)	0.126 (10)	0.028 (4)	−0.013 (5)	−0.002 (3)	−0.001 (5)
C70	0.033 (4)	0.077 (6)	0.031 (4)	−0.003 (4)	−0.004 (3)	0.008 (4)
C71	0.018 (3)	0.045 (4)	0.031 (3)	−0.009 (3)	0.005 (2)	−0.003 (3)
C72	0.019 (3)	0.056 (5)	0.034 (4)	−0.009 (3)	0.008 (3)	0.006 (3)
O11	0.108 (3)	0.127 (4)	0.101 (3)	−0.076 (3)	−0.014 (2)	0.022 (3)
O12	0.108 (3)	0.127 (4)	0.101 (3)	−0.076 (3)	−0.014 (2)	0.022 (3)
O13	0.108 (3)	0.127 (4)	0.101 (3)	−0.076 (3)	−0.014 (2)	0.022 (3)
N5	0.108 (3)	0.127 (4)	0.101 (3)	−0.076 (3)	−0.014 (2)	0.022 (3)
O1W	0.042 (2)	0.019 (2)	0.050 (3)	−0.0106 (19)	0.003 (2)	−0.0084 (19)
O2W	0.090 (4)	0.067 (4)	0.049 (3)	−0.026 (4)	0.014 (3)	−0.016 (3)

### Geometric parameters (Å, °)

**Table:**

Er1—O1	2.470 (4)	C44—C52	1.417 (8)
Er1—O2	2.376 (4)	C44—C45	1.431 (8)
Er1—O3W	2.358 (3)	C45—C46	1.331 (9)
Er1—O4	2.372 (3)	C46—C47	1.437 (9)
Er1—O5	2.399 (3)	C47—C51	1.399 (8)
Er1—O6	2.366 (4)	C47—C48	1.399 (9)
Er1—O7	2.433 (4)	C48—C49	1.355 (10)
Er1—N1	2.461 (4)	C49—C50	1.396 (9)
Er1—N2	2.489 (4)	C51—C52	1.457 (8)
O1—N8	1.264 (7)	C23—H23	0.9300
O2—N8	1.264 (6)	C24—H24	0.9300
O3—N8	1.221 (6)	C26—H26	0.9300
O4—C21	1.259 (6)	C27—H27	0.9300
O5—C21	1.289 (7)	C33—H33	0.9300
O6—C31	1.273 (7)	C34—H34	0.9300
O7—C31	1.279 (7)	C36—H36	0.9300
O8—C25	1.346 (7)	C37—H37	0.9300
O9—C35	1.356 (10)	C41—H41	0.9300
O3W—H3B	0.8200	C42—H42	0.9300
O3W—H3A	0.8200	C43—H43	0.9300
O8—H8	0.8200	C45—H45	0.9300
O9—H9	0.8200	C46—H46	0.9300
O11—N5	1.274 (15)	C48—H48	0.9300
O12—N5	1.217 (14)	C49—H49	0.9300
O13—N5	1.272 (16)	C50—H50	0.9300
O1W—H1A	0.8200	C81—C82	1.368 (9)
O1W—H1B	0.8200	C81—C81^i^	1.447 (8)
N1—C41	1.333 (7)	C81—C86	1.381 (9)
N1—C52	1.353 (7)	C82—C83	1.389 (10)
N2—C51	1.365 (7)	C82—C87	1.504 (17)
N2—C50	1.332 (7)	C83—C84	1.345 (12)
O2W—H2B	0.8200	C84—C85	1.350 (13)
O2W—H2A	0.8200	C85—C86	1.381 (10)
N6—C81	1.368 (9)	C87—C88	1.31 (2)
N6—C87	1.504 (17)	C83—H83	0.9300
N6—C83	1.389 (10)	C84—H84	0.9300
N7—C85	1.381 (10)	C85—H85	0.9300
N7—C81	1.381 (9)	C87—H87	0.9300
N3—C61	1.312 (10)	C88—H88	0.9300
N3—C72	1.350 (10)	C61—C62	1.380 (15)
N4—C71	1.328 (10)	C62—C63	1.325 (15)
N4—C70	1.326 (10)	C63—C64	1.400 (12)
N4—H4	0.8600	C64—C72	1.383 (11)
C21—C22	1.470 (8)	C64—C65	1.444 (13)
C22—C27	1.403 (8)	C65—C66	1.346 (16)
C22—C23	1.391 (8)	C66—C67	1.420 (14)
C23—C24	1.371 (8)	C67—C68	1.371 (11)
C24—C25	1.384 (9)	C67—C71	1.410 (11)
C25—C26	1.394 (8)	C68—C69	1.356 (17)
C26—C27	1.371 (8)	C69—C70	1.358 (14)
C31—C32	1.474 (8)	C71—C72	1.448 (8)
C32—C37	1.381 (8)	C61—H61	0.9300
C32—C33	1.396 (8)	C62—H62	0.9300
C33—C34	1.381 (9)	C63—H63	0.9300
C34—C35	1.375 (10)	C65—H65	0.9300
C35—C36	1.396 (12)	C66—H66	0.9300
C36—C37	1.371 (10)	C68—H68	0.9300
C41—C42	1.408 (8)	C69—H69	0.9300
C42—C43	1.355 (8)	C70—H70	0.9300
C43—C44	1.395 (8)		
			
O1—Er1—O2	52.34 (14)	N1—C41—C42	123.0 (5)
O1—Er1—O3W	144.85 (13)	C41—C42—C43	118.6 (5)
O1—Er1—O4	120.59 (13)	C42—C43—C44	120.7 (5)
O1—Er1—O5	130.28 (13)	C43—C44—C45	123.8 (5)
O1—Er1—O6	70.36 (13)	C43—C44—C52	116.9 (5)
O1—Er1—O7	111.53 (13)	C45—C44—C52	119.3 (5)
O1—Er1—N1	72.55 (13)	C44—C45—C46	121.4 (6)
O1—Er1—N2	68.63 (14)	C45—C46—C47	121.7 (6)
O1—Er1—C21	129.79 (16)	C46—C47—C51	119.0 (6)
O1—Er1—C31	91.78 (15)	C48—C47—C51	117.6 (6)
O2—Er1—O3W	147.75 (12)	C46—C47—C48	123.5 (5)
O2—Er1—O4	75.78 (12)	C47—C48—C49	120.1 (6)
O2—Er1—O5	83.49 (13)	C48—C49—C50	119.1 (6)
O2—Er1—O6	75.18 (13)	N2—C50—C49	123.0 (6)
O2—Er1—O7	128.27 (12)	N2—C51—C47	122.5 (5)
O2—Er1—N1	84.17 (14)	N2—C51—C52	117.6 (5)
O2—Er1—N2	119.87 (14)	C47—C51—C52	119.8 (5)
O2—Er1—C21	77.53 (15)	C44—C52—C51	118.7 (5)
O2—Er1—C31	101.38 (15)	N1—C52—C51	118.5 (5)
O3W—Er1—O4	72.86 (12)	N1—C52—C44	122.8 (5)
O3W—Er1—O5	84.64 (11)	C22—C23—H23	120.00
O3W—Er1—O6	130.89 (12)	C24—C23—H23	120.00
O3W—Er1—O7	77.04 (12)	C25—C24—H24	119.00
O3W—Er1—N1	81.08 (13)	C23—C24—H24	119.00
O3W—Er1—N2	80.02 (12)	C25—C26—H26	120.00
O3W—Er1—C21	78.02 (14)	C27—C26—H26	120.00
O3W—Er1—C31	103.94 (15)	C22—C27—H27	120.00
O4—Er1—O5	54.83 (12)	C26—C27—H27	120.00
O4—Er1—O6	126.38 (13)	C34—C33—H33	120.00
O4—Er1—O7	123.64 (12)	C32—C33—H33	120.00
O4—Er1—N1	75.77 (12)	C33—C34—H34	120.00
O4—Er1—N2	136.77 (12)	C35—C34—H34	120.00
O4—Er1—C21	27.04 (14)	C35—C36—H36	120.00
O4—Er1—C31	128.77 (14)	C37—C36—H36	120.00
O5—Er1—O6	77.85 (13)	C36—C37—H37	120.00
O5—Er1—O7	76.12 (11)	C32—C37—H37	120.00
O5—Er1—N1	130.59 (12)	N1—C41—H41	119.00
O5—Er1—N2	154.27 (14)	C42—C41—H41	118.00
O5—Er1—C21	27.81 (14)	C43—C42—H42	121.00
O5—Er1—C31	73.95 (14)	C41—C42—H42	121.00
O6—Er1—O7	54.37 (12)	C44—C43—H43	120.00
O6—Er1—N1	142.83 (13)	C42—C43—H43	120.00
O6—Er1—N2	96.83 (13)	C46—C45—H45	119.00
O6—Er1—C21	102.45 (15)	C44—C45—H45	119.00
O6—Er1—C31	27.09 (15)	C47—C46—H46	119.00
O7—Er1—N1	143.43 (13)	C45—C46—H46	119.00
O7—Er1—N2	80.37 (13)	C49—C48—H48	120.00
O7—Er1—C21	100.89 (14)	C47—C48—H48	120.00
O7—Er1—C31	27.34 (14)	C48—C49—H49	120.00
N1—Er1—N2	67.14 (14)	C50—C49—H49	121.00
N1—Er1—C21	102.79 (15)	C49—C50—H50	118.00
N1—Er1—C31	155.46 (15)	N2—C50—H50	119.00
N2—Er1—C21	157.08 (15)	C81^i^—C81—C82	118.6 (6)
N2—Er1—C31	89.81 (15)	N7—C81—C82	122.5 (5)
C21—Er1—C31	101.75 (16)	N7—C81—C81^i^	118.9 (6)
Er1—O1—N8	93.5 (3)	C82—C81—C86	122.5 (5)
Er1—O2—N8	98.0 (3)	N6—C81—C81^i^	118.6 (6)
Er1—O4—C21	94.0 (3)	N6—C81—C86	122.5 (5)
Er1—O5—C21	92.0 (3)	C81^i^—C81—C86	118.9 (6)
Er1—O6—C31	95.1 (3)	N6—C81—N7	122.5 (5)
Er1—O7—C31	91.8 (3)	C83—C82—C87	120.5 (8)
H3A—O3W—H3B	110.00	C81—C82—C83	116.7 (6)
Er1—O3W—H3B	110.00	C81—C82—C87	122.7 (8)
Er1—O3W—H3A	118.00	N6—C83—C84	122.4 (8)
C25—O8—H8	109.00	C82—C83—C84	122.4 (8)
C35—O9—H9	109.00	C83—C84—C85	119.4 (7)
H1A—O1W—H1B	108.00	C84—C85—C86	121.7 (8)
C41—N1—C52	117.8 (5)	N7—C85—C84	121.7 (8)
Er1—N1—C41	124.3 (3)	C81—C86—C85	117.4 (6)
Er1—N1—C52	117.7 (3)	C82—C87—C88	117.9 (13)
C50—N2—C51	117.7 (5)	N6—C87—C88	117.9 (13)
Er1—N2—C50	125.0 (4)	C82—C83—H83	119.00
Er1—N2—C51	116.3 (3)	C84—C83—H83	119.00
O1—N8—O2	115.5 (4)	N6—C83—H83	119.00
O1—N8—O3	121.9 (5)	C85—C84—H84	120.00
O2—N8—O3	122.5 (5)	C83—C84—H84	120.00
H2A—O2W—H2B	108.00	C84—C85—H85	119.00
C81—N6—C87	122.7 (8)	N7—C85—H85	119.00
C81—N6—C83	116.7 (6)	C86—C85—H85	119.00
C83—N6—C87	120.5 (8)	C88—C87—H87	121.00
C81—N7—C85	117.4 (6)	N6—C87—H87	121.00
C61—N3—C72	116.4 (8)	C82—C87—H87	121.00
C70—N4—C71	120.9 (7)	C87—C88—H88	120.00
C71—N4—H4	120.00	N3—C61—C62	124.2 (9)
C70—N4—H4	119.00	C61—C62—C63	119.4 (9)
O12—N5—O13	117.0 (11)	C62—C63—C64	119.3 (10)
O11—N5—O13	120.0 (11)	C63—C64—C65	123.3 (9)
O11—N5—O12	122.7 (12)	C63—C64—C72	117.6 (8)
Er1—C21—C22	177.2 (4)	C65—C64—C72	119.1 (7)
O4—C21—O5	119.1 (5)	C64—C65—C66	120.9 (9)
O4—C21—C22	120.6 (5)	C65—C66—C67	121.7 (10)
O5—C21—C22	120.3 (5)	C68—C67—C71	117.4 (9)
Er1—C21—O5	60.2 (3)	C66—C67—C71	118.9 (8)
Er1—C21—O4	59.0 (3)	C66—C67—C68	123.6 (9)
C21—C22—C27	120.9 (5)	C67—C68—C69	120.9 (9)
C23—C22—C27	119.1 (5)	C68—C69—C70	119.0 (8)
C21—C22—C23	120.0 (5)	N4—C70—C69	121.6 (9)
C22—C23—C24	120.1 (5)	N4—C71—C72	120.4 (6)
C23—C24—C25	121.1 (6)	C67—C71—C72	119.4 (7)
C24—C25—C26	119.0 (5)	N4—C71—C67	120.2 (6)
O8—C25—C24	118.0 (5)	N3—C72—C71	116.9 (6)
O8—C25—C26	123.1 (5)	C64—C72—C71	120.1 (6)
C25—C26—C27	120.5 (5)	N3—C72—C64	123.0 (6)
C22—C27—C26	120.2 (5)	N3—C61—H61	118.00
Er1—C31—C32	174.6 (4)	C62—C61—H61	118.00
Er1—C31—O6	57.9 (3)	C61—C62—H62	120.00
O6—C31—C32	119.9 (5)	C63—C62—H62	120.00
Er1—C31—O7	60.9 (3)	C64—C63—H63	120.00
O7—C31—C32	121.6 (5)	C62—C63—H63	120.00
O6—C31—O7	118.5 (5)	C66—C65—H65	120.00
C33—C32—C37	118.7 (5)	C64—C65—H65	120.00
C31—C32—C37	120.2 (6)	C65—C66—H66	119.00
C31—C32—C33	121.1 (5)	C67—C66—H66	119.00
C32—C33—C34	120.8 (5)	C67—C68—H68	120.00
C33—C34—C35	119.7 (7)	C69—C68—H68	120.00
C34—C35—C36	119.9 (7)	C70—C69—H69	120.00
O9—C35—C36	117.9 (7)	C68—C69—H69	121.00
O9—C35—C34	122.1 (7)	C69—C70—H70	119.00
C35—C36—C37	120.0 (7)	N4—C70—H70	119.00
C32—C37—C36	120.9 (7)		
			
O2—Er1—O1—N8	4.8 (3)	Er1—O6—C31—O7	5.4 (5)
O3W—Er1—O1—N8	144.2 (3)	Er1—O6—C31—C32	−174.3 (4)
O4—Er1—O1—N8	40.0 (3)	Er1—O7—C31—C32	174.4 (5)
O5—Er1—O1—N8	−27.9 (4)	Er1—O7—C31—O6	−5.2 (5)
O6—Er1—O1—N8	−81.4 (3)	C52—N1—C41—C42	0.6 (8)
O7—Er1—O1—N8	−117.7 (3)	Er1—N1—C52—C44	−172.0 (4)
N1—Er1—O1—N8	101.0 (3)	Er1—N1—C52—C51	9.3 (6)
N2—Er1—O1—N8	172.7 (3)	C41—N1—C52—C44	2.4 (8)
C21—Er1—O1—N8	8.7 (4)	Er1—N1—C41—C42	174.6 (4)
C31—Er1—O1—N8	−98.2 (3)	C41—N1—C52—C51	−176.4 (5)
O1—Er1—O2—N8	−4.8 (3)	C50—N2—C51—C47	−1.6 (8)
O3W—Er1—O2—N8	−140.2 (3)	C50—N2—C51—C52	175.3 (5)
O4—Er1—O2—N8	−154.0 (3)	Er1—N2—C51—C52	−15.8 (6)
O5—Er1—O2—N8	150.7 (3)	Er1—N2—C50—C49	−165.8 (4)
O6—Er1—O2—N8	71.6 (3)	C51—N2—C50—C49	2.1 (8)
O7—Er1—O2—N8	84.1 (3)	Er1—N2—C51—C47	167.3 (4)
N1—Er1—O2—N8	−77.2 (3)	C83—N6—C87—C88	−179.0 (12)
N2—Er1—O2—N8	−17.8 (3)	C83—N6—C81—C86	0.4 (11)
C21—Er1—O2—N8	178.3 (3)	C83—N6—C81—C81^i^	179.9 (7)
C31—Er1—O2—N8	78.6 (3)	C87—N6—C81—N7	177.0 (9)
O1—Er1—O4—C21	−118.5 (3)	C87—N6—C81—C86	177.0 (9)
O2—Er1—O4—C21	−90.4 (3)	C87—N6—C81—C81^i^	−3.4 (12)
O3W—Er1—O4—C21	97.3 (3)	C81—N6—C83—C84	0.5 (12)
O5—Er1—O4—C21	1.7 (3)	C83—N6—C81—N7	0.4 (11)
O6—Er1—O4—C21	−31.3 (4)	C81—N6—C87—C88	4.5 (18)
O7—Er1—O4—C21	36.4 (3)	C87—N6—C83—C84	−176.3 (10)
N1—Er1—O4—C21	−177.9 (3)	C85—N7—C81—C81^i^	179.6 (7)
N2—Er1—O4—C21	150.9 (3)	C81—N7—C85—C84	0.5 (12)
C31—Er1—O4—C21	2.9 (4)	C85—N7—C81—C82	−0.8 (11)
O1—Er1—O5—C21	101.0 (3)	C85—N7—C81—N6	−0.8 (11)
O2—Er1—O5—C21	75.6 (3)	C61—N3—C72—C64	−2.2 (11)
O3W—Er1—O5—C21	−74.4 (3)	C72—N3—C61—C62	−0.2 (12)
O4—Er1—O5—C21	−1.6 (3)	C61—N3—C72—C71	177.9 (7)
O6—Er1—O5—C21	151.8 (3)	C71—N4—C70—C69	−0.8 (12)
O7—Er1—O5—C21	−152.4 (3)	C70—N4—C71—C67	−0.2 (11)
N1—Er1—O5—C21	−1.0 (4)	C70—N4—C71—C72	−178.6 (7)
N2—Er1—O5—C21	−127.8 (4)	O5—C21—C22—C23	−179.2 (5)
C31—Er1—O5—C21	179.4 (3)	O4—C21—C22—C27	−176.5 (5)
O1—Er1—O6—C31	−140.4 (3)	O5—C21—C22—C27	3.2 (8)
O2—Er1—O6—C31	164.8 (3)	O4—C21—C22—C23	1.2 (8)
O3W—Er1—O6—C31	6.7 (4)	C21—C22—C23—C24	−177.9 (6)
O4—Er1—O6—C31	105.5 (3)	C23—C22—C27—C26	0.2 (9)
O5—Er1—O6—C31	78.5 (3)	C21—C22—C27—C26	177.9 (5)
O7—Er1—O6—C31	−3.1 (3)	C27—C22—C23—C24	−0.2 (9)
N1—Er1—O6—C31	−136.7 (3)	C22—C23—C24—C25	−0.3 (9)
N2—Er1—O6—C31	−76.0 (3)	C23—C24—C25—C26	0.8 (9)
C21—Er1—O6—C31	91.5 (3)	C23—C24—C25—O8	−179.0 (6)
O1—Er1—O7—C31	46.4 (3)	O8—C25—C26—C27	179.0 (5)
O2—Er1—O7—C31	−11.9 (3)	C24—C25—C26—C27	−0.8 (9)
O3W—Er1—O7—C31	−169.4 (3)	C25—C26—C27—C22	0.3 (9)
O4—Er1—O7—C31	−110.5 (3)	O6—C31—C32—C37	2.9 (8)
O5—Er1—O7—C31	−81.8 (3)	O6—C31—C32—C33	−176.3 (5)
O6—Er1—O7—C31	3.0 (3)	O7—C31—C32—C33	4.1 (8)
N1—Er1—O7—C31	135.8 (3)	O7—C31—C32—C37	−176.7 (5)
N2—Er1—O7—C31	108.7 (3)	C31—C32—C33—C34	178.5 (5)
C21—Er1—O7—C31	−94.6 (3)	C33—C32—C37—C36	0.2 (9)
O1—Er1—N1—C41	−112.5 (4)	C31—C32—C37—C36	−179.1 (6)
O1—Er1—N1—C52	61.5 (4)	C37—C32—C33—C34	−0.8 (8)
O2—Er1—N1—C41	−60.2 (4)	C32—C33—C34—C35	0.6 (9)
O2—Er1—N1—C52	113.7 (4)	C33—C34—C35—O9	−177.7 (7)
O3W—Er1—N1—C41	91.0 (4)	C33—C34—C35—C36	0.3 (11)
O3W—Er1—N1—C52	−95.0 (4)	O9—C35—C36—C37	177.2 (7)
O4—Er1—N1—C41	16.6 (4)	C34—C35—C36—C37	−0.8 (12)
O4—Er1—N1—C52	−169.5 (4)	C35—C36—C37—C32	0.6 (11)
O5—Er1—N1—C41	16.0 (5)	N1—C41—C42—C43	−2.0 (9)
O5—Er1—N1—C52	−170.0 (3)	C41—C42—C43—C44	0.4 (9)
O6—Er1—N1—C41	−116.2 (4)	C42—C43—C44—C45	−178.3 (6)
O6—Er1—N1—C52	57.8 (5)	C42—C43—C44—C52	2.3 (8)
O7—Er1—N1—C41	144.7 (4)	C43—C44—C52—C51	174.9 (5)
O7—Er1—N1—C52	−41.4 (5)	C45—C44—C52—C51	−4.5 (8)
N2—Er1—N1—C41	173.9 (5)	C45—C44—C52—N1	176.7 (5)
N2—Er1—N1—C52	−12.2 (4)	C43—C44—C45—C46	−177.6 (6)
C21—Er1—N1—C41	15.6 (4)	C43—C44—C52—N1	−3.8 (8)
C21—Er1—N1—C52	−170.5 (4)	C52—C44—C45—C46	1.9 (9)
C31—Er1—N1—C41	−164.9 (4)	C44—C45—C46—C47	2.7 (10)
C31—Er1—N1—C52	9.0 (6)	C45—C46—C47—C51	−4.5 (9)
O1—Er1—N2—C50	103.0 (4)	C45—C46—C47—C48	175.1 (6)
O1—Er1—N2—C51	−65.0 (4)	C48—C47—C51—C52	−178.0 (5)
O2—Er1—N2—C50	114.1 (4)	C46—C47—C51—N2	178.4 (5)
O2—Er1—N2—C51	−53.9 (4)	C46—C47—C48—C49	−176.1 (6)
O3W—Er1—N2—C50	−93.2 (4)	C51—C47—C48—C49	3.5 (9)
O3W—Er1—N2—C51	98.9 (4)	C46—C47—C51—C52	1.6 (8)
O4—Er1—N2—C50	−144.6 (4)	C48—C47—C51—N2	−1.1 (9)
O4—Er1—N2—C51	47.5 (4)	C47—C48—C49—C50	−3.0 (9)
O5—Er1—N2—C50	−38.9 (6)	C48—C49—C50—N2	0.2 (9)
O5—Er1—N2—C51	153.2 (3)	N2—C51—C52—N1	4.6 (8)
O6—Er1—N2—C50	37.3 (4)	N2—C51—C52—C44	−174.2 (5)
O6—Er1—N2—C51	−130.7 (4)	C47—C51—C52—C44	2.8 (8)
O7—Er1—N2—C50	−14.8 (4)	C47—C51—C52—N1	−178.4 (5)
O7—Er1—N2—C51	177.3 (4)	C86—C81—C82—C83	0.4 (11)
N1—Er1—N2—C50	−177.6 (5)	C86—C81—C82—C87	177.0 (9)
N1—Er1—N2—C51	14.4 (3)	N7—C81—C82—C87	177.0 (9)
C21—Er1—N2—C50	−110.0 (5)	N6—C81—C81^i^—N6^i^	−180.0 (7)
C21—Er1—N2—C51	82.1 (6)	N6—C81—C81^i^—N7^i^	0.4 (10)
C31—Er1—N2—C50	11.0 (4)	C81^i^—C81—C82—C83	179.9 (7)
C31—Er1—N2—C51	−156.9 (4)	C81^i^—C81—C82—C87	−3.4 (12)
O1—Er1—C21—O4	79.9 (3)	N6—C81—C86—C85	−0.8 (11)
O1—Er1—C21—O5	−103.0 (3)	C82—C81—C86—C85	−0.8 (11)
O2—Er1—C21—O4	83.1 (3)	C81^i^—C81—C86—C85	179.6 (7)
O2—Er1—C21—O5	−99.8 (3)	C86—C81—C81^i^—N7^i^	−180.0 (7)
O3W—Er1—C21—O4	−75.7 (3)	N7—C81—C82—C83	0.4 (11)
O3W—Er1—C21—O5	101.4 (3)	C82—C81—C81^i^—N6^i^	−180.0 (7)
O4—Er1—C21—O5	177.1 (5)	N7—C81—C81^i^—N6^i^	−0.4 (10)
O5—Er1—C21—O4	−177.1 (5)	N7—C81—C81^i^—N7^i^	−180.0 (7)
O6—Er1—C21—O4	154.6 (3)	C82—C81—C81^i^—N7^i^	0.4 (10)
O6—Er1—C21—O5	−28.3 (3)	C86—C81—C81^i^—N6^i^	−0.4 (10)
O7—Er1—C21—O4	−149.8 (3)	C87—C82—C83—C84	−176.3 (10)
O7—Er1—C21—O5	27.3 (3)	C81—C82—C83—C84	0.5 (12)
N1—Er1—C21—O4	2.1 (3)	C81—C82—C87—C88	4.5 (18)
N1—Er1—C21—O5	179.2 (3)	C83—C82—C87—C88	−179.0 (12)
N2—Er1—C21—O4	−58.8 (6)	N6—C83—C84—C85	−0.8 (14)
N2—Er1—C21—O5	118.3 (4)	C82—C83—C84—C85	−0.8 (14)
C31—Er1—C21—O4	−177.7 (3)	C83—C84—C85—C86	0.3 (14)
C31—Er1—C21—O5	−0.6 (3)	C83—C84—C85—N7	0.3 (14)
O1—Er1—C31—O6	36.9 (3)	C84—C85—C86—C81	0.5 (12)
O1—Er1—C31—O7	−137.7 (3)	N3—C61—C62—C63	1.9 (14)
O2—Er1—C31—O6	−15.0 (3)	C61—C62—C63—C64	−1.1 (13)
O2—Er1—C31—O7	170.5 (3)	C62—C63—C64—C65	179.8 (9)
O3W—Er1—C31—O6	−174.8 (3)	C62—C63—C64—C72	−1.1 (12)
O3W—Er1—C31—O7	10.6 (3)	C63—C64—C65—C66	177.9 (9)
O4—Er1—C31—O6	−95.8 (3)	C72—C64—C65—C66	−1.2 (13)
O4—Er1—C31—O7	89.7 (3)	C63—C64—C72—N3	2.9 (11)
O5—Er1—C31—O6	−94.7 (3)	C63—C64—C72—C71	−177.3 (7)
O5—Er1—C31—O7	90.7 (3)	C65—C64—C72—N3	−178.0 (7)
O6—Er1—C31—O7	−174.6 (5)	C65—C64—C72—C71	1.9 (11)
O7—Er1—C31—O6	174.6 (5)	C64—C65—C66—C67	0.2 (15)
N1—Er1—C31—O6	86.1 (5)	C65—C66—C67—C68	−178.3 (10)
N1—Er1—C31—O7	−88.5 (5)	C65—C66—C67—C71	0.1 (14)
N2—Er1—C31—O6	105.5 (3)	C66—C67—C68—C69	178.4 (10)
N2—Er1—C31—O7	−69.1 (3)	C71—C67—C68—C69	0.0 (14)
C21—Er1—C31—O6	−94.4 (3)	C66—C67—C71—N4	−177.9 (8)
C21—Er1—C31—O7	91.0 (3)	C66—C67—C71—C72	0.5 (11)
Er1—O1—N8—O3	173.3 (5)	C68—C67—C71—N4	0.6 (11)
Er1—O1—N8—O2	−7.9 (4)	C68—C67—C71—C72	179.0 (8)
Er1—O2—N8—O3	−173.0 (5)	C67—C68—C69—C70	−0.9 (15)
Er1—O2—N8—O1	8.3 (5)	C68—C69—C70—N4	1.3 (14)
Er1—O4—C21—O5	−2.9 (5)	N4—C71—C72—N3	−3.3 (10)
Er1—O4—C21—C22	176.8 (5)	N4—C71—C72—C64	176.9 (7)
Er1—O5—C21—C22	−176.8 (5)	C67—C71—C72—N3	178.3 (7)
Er1—O5—C21—O4	2.9 (5)	C67—C71—C72—C64	−1.5 (10)

Symmetry codes: (i) −*x*+2, −*y*, −*z*+1.

### Hydrogen-bond geometry (Å, °)

**Table:**

Cg4 is the centroid of the C32–C37 ring.

**Table:**

*D*—H···*A*	*D*—H	H···*A*	*D*···*A*	*D*—H···*A*
O1W—H1A···O2^ii^	0.82	2.55	3.270 (6)	148
O1W—H1A···O6^ii^	0.82	2.22	2.875 (6)	137
O1W—H1B···O12^ii^	0.82	2.06	2.872 (10)	172
O2W—H2A···O9^iii^	0.82	1.94	2.737 (8)	164
O2W—H2B···O11^ii^	0.82	2.01	2.789 (10)	157
O3W—H3A···O5^iv^	0.82	1.90	2.671 (5)	155
O3W—H3B···O7^iv^	0.82	2.16	2.836 (5)	140
N4—H4···O2W	0.86	1.91	2.725 (9)	157
O8—H8···O1W^v^	0.82	1.87	2.659 (6)	160
O9—H9···O11^vi^	0.82	2.09	2.803 (12)	145
O9—H9···O12^vi^	0.82	2.43	3.172 (12)	151
C34—H34···O3^vi^	0.93	2.43	3.256 (8)	148
C48—H48···O12^vii^	0.93	2.27	3.046 (11)	141
C62—H62···O1^viii^	0.93	2.55	3.387 (10)	150
C70—H70···O13^ii^	0.93	2.35	3.244 (13)	161
C83—H83···Cg4^iii^	0.93	2.78	3.628 (9)	152

Symmetry codes: (ii) −*x*+1, −*y*+1, −*z*+1; (iii) *x*+1, *y*, *z*; (iv) −*x*, −*y*+1, −*z*; (v) *x*−1, *y*+1, *z*−1; (vi) *x*−1, *y*, *z*; (vii) *x*, *y*−1, *z*; (viii) *x*, *y*+1, *z*.
